# Associations between residential greenness and self-reported heart disease in Sri Lankan men: A cross-sectional study

**DOI:** 10.1371/journal.pone.0252382

**Published:** 2021-05-28

**Authors:** J. Padmaka Silva, Ankur Singh, Brian Oldenburg, Wasantha Gunathunga, A. M. A. A. P. Alagiyawanna, Suzanne Mavoa

**Affiliations:** 1 Office of the Regional Director of Health Services, Monaragala, Sri Lanka; 2 Melbourne School of Population & Global Health, The University of Melbourne, Melbourne, Australia; 3 Department of Community Medicine, Faculty of Medicine, University of Colombo, Colombo, Sri Lanka; 4 Health Promotion Bureau, Ministry of Health, Colombo, Sri Lanka; Kaohsiung Medical University, TAIWAN

## Abstract

Cardiovascular diseases (CVDs) are major contributors to morbidity and mortality in lower-middle-income countries (LMICs). Features of the natural environment, such as greenness, are a potential, modifiable determinant of CVD, yet there is a lack of evidence, particularly in LMICs. Our study investigated associations between residential greenness, measured using the Normalized Difference Vegetation Index (NDVI), and self-reported heart disease in 5268 Sri Lankan men aged 34 to 55 years. Multivariable logistic regression models were fitted to examine associations between mean NDVI within 100 m, 400 m, 800 m, 1600 m, and 2000 m of the residential address, adjusting for age, marital status, income, education, alcohol consumption, smoking and road length. Fully adjusted models showed that a 0.1 increase in mean NDVI was associated with lower odds of heart disease when using the 400 m (OR: 0.80; 95% CI: 0.64, 1.00), 800 m (OR: 0.85; 95% CI: 0.63, 1.14), and 2000 m (OR: 0.74; 95% CI: 0.48, 1.13) buffers. Further research in different contexts, and with improved outcome measures, is needed to confirm relationships between residential greenness and heart disease in rural areas and in LMICs.

## Introduction

Eighty percent of all noncommunicable disease (NCD) related deaths now occur in lower- middle-income countries (LMICs) [1] with cardiovascular diseases a major contributor to this burden [2]. This rise in NCDs has occurred at the same time as rapid urbanization, which brings with it changes to the environments that people are exposed to (e.g., increased air pollution, decreased places and opportunities to be physical active, decreased vegetation), and these environmental risk factors have been linked to NCDs and behavioral risk factors [3]. However, there are many evidence gaps. Much of the evidence relating to environmental determinants of NCDs comes from high-income countries [1,2,4]. Further, context matters and there are demonstrable differences and spatial patterns in relationships between urbanization and various NCDs across different populations and contexts [3], making it important to test and replicate research findings in different LMICs.

Natural environments, particularly vegetation levels (i.e., ‘greenness’) and green spaces (i.e., dedicated green areas such as parks and sports fields) have been shown to have a range of health benefits. Findings from systematic reviews conclude that exposure to green spaces and greater levels of greenness are associated with lower mortality and greater physiological and psychological health benefits [5–9]. However, most research is from high-income countries with little evidence from LMICs in Asia, Africa, and South America [10]. In a recent systematic review and meta-analysis on greenspace exposure and health outcomes, only 10 out of 143 studies were from Asia, Africa, or South America [10]. Of these, only six were from LMICs (five from China and one from Brazil) withfindings from these studies demonstrated health benefits from green spaces [10]. Research published since has shown that higher greenness was associated with lower incidence of depression among middle-income group participants in South Africa [11], reduced hypertension and cardiometabolic biomarkers in India [12], and lower reduced cardiovascular risk factors in Sub-Saharan Africa [13]. However, evidence from different contexts such as non-temperate LMICs is still [14].

Our study addresses this important research gap by investigating whether neighborhood greenness, an emerging environmental risk factor in high-income countries [5–8], is associated with self-report cardiovascular morbidity among 5,268 working-age men in Ingiriya, Sri Lanka. Sri Lanka, like other LMICs, is experiencing rapid urbanization and an increase in NCD burden [15], which is further straining an already stretched health system and economy [16]. Cardiovascular diseases have been the leading cause of death in Sri Lanka over the past few decades [17]. This increase is partly due to a rapidly urbanizing population, and subsequent changes in lifestyle behaviors and environments, both of which are cardiovascular risk factors. Further, the prevalence of and risk factors for most NCDs are higher among Sri Lankan men, compared to women [18].

## Methods

### Study area

Ingiriya is a rural agricultural town located in the district of Kalutara in the Western Province of Sri Lanka. It has a population of approximately 60,000, and a population density of 5.5 people per hectare [19]. In comparison, Colombo, the capital city of Sri Lanka has a population density of 155.7 people per hectare [19]. Many people work in agriculture without the assistance of machinery and are therefore physically active. For instance, a nationally representative study of Sri Lankan adults found that 60% of Sri Lankan adults were highly active, with rural adults significantly more active than urban adults [20].

**Fig 1:**
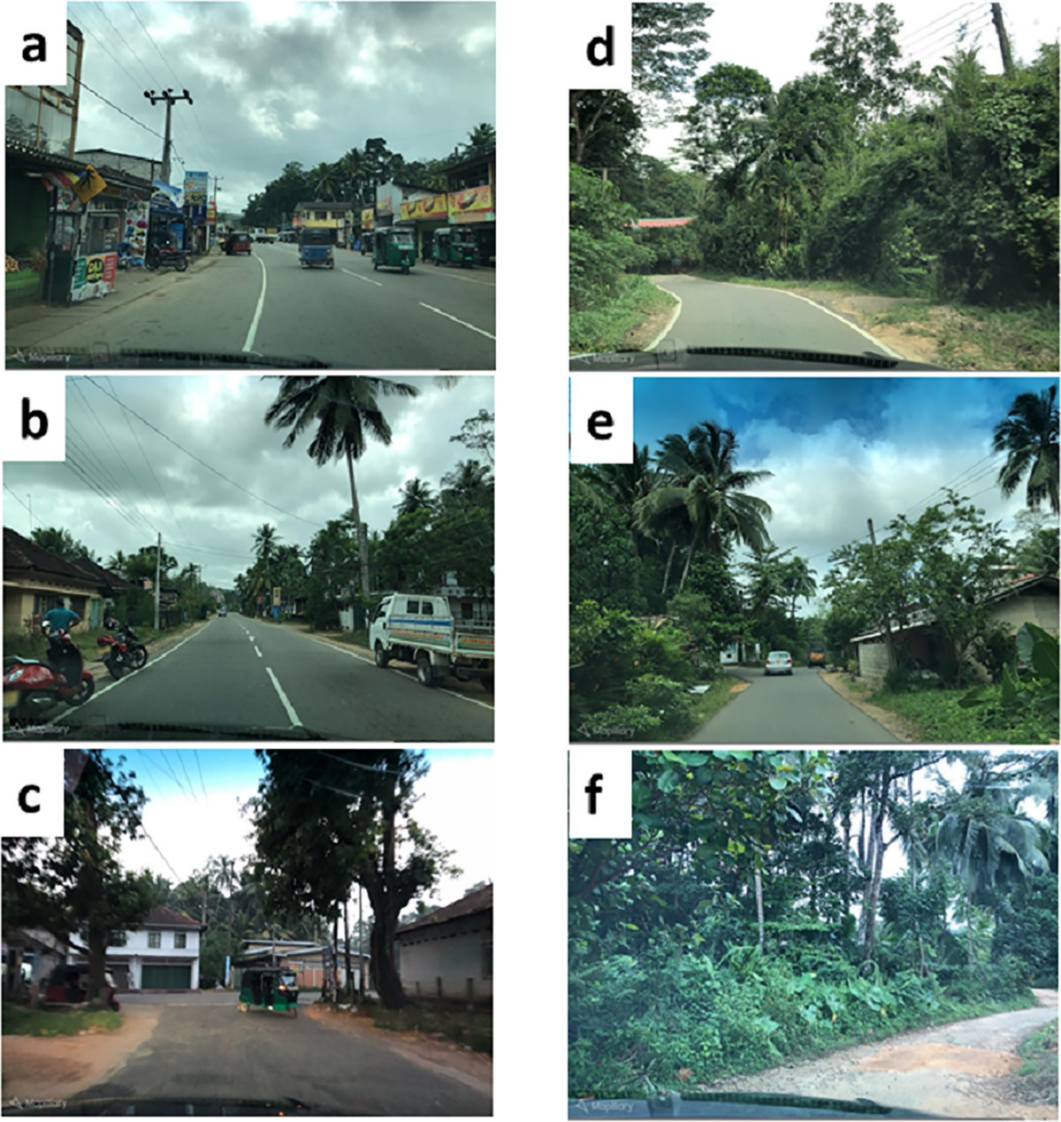
Typical vegetation. a) town centre on a main trunk road, b) a main trunk road, c) a town area (next to a main trunk road), d) main road, e) road leading to residences, f) minor road/track leading to residences. Source: Mapillary (https://www.mapillary.com/). Accessed 19 February 2021. All images CC BY-SA 4.0. Due to lack of freely available images in the study area, the images in this figure are from nearby areas and are representative of the study area. These images have been visually checked against images from the study area from Google StreetView.

Ingiriya has very high levels of vegetation including forests, and rubber, tea, and coconut plantations, and rice paddies [21]. The town centre is on the main road, which runs through the region (similar to Fig 1A). Examples of vegetation typical in different residential locations in the study area are shown in Fig 1.

### Household survey

This study was a secondary analysis of a dataset derived from a household survey of all working-age men (34 to 55 years) residing in the study area. The survey was conducted from May to December 2012 by the first author and five trained undergraduate students. Households in the area were identified with assistance from the Medical Officer and Public Health Midwives who are grass roots public health workers with an in-depth knowledge of the area. Data collectors approached these households (n = 5,500) and enrolled working men in the study. If no one was home, the household was revisited a second time. A total of 5,402 participants were enrolled (response rate = 97.5%). The survey was conducted in-person at participants’ homes and collected socio-demographic data, and self-reported health and behavior data including cardiovascular disease, mental health status, and unhealthy behaviors (e.g., smoking, alcohol consumption). Details of the survey are reported elsewhere [22].

At the time of the survey, the geographic coordinates (latitude and longitude) of the residence of each participant were recorded using GPS equipment. This approach provides more accurate location data than other methods typically used to assess residential location. For instance, while researchers often use administrative areas to represent residential locations and exposures, these areas are often not ideal for assessment of individual exposures [23]. Our direct GPS measurement avoids the common approach of geocoding addresses based on address text which can lead to inaccurate locations within a plot of land [24]; a potentially greater problem in our study area which is rural with large land parcels. Of the 5,402 participants enrolled, 5,268 responded to questions related to self-reported heart diseases.

Ethics approval was granted by the Ethics Committee of the Faculty of Medicine Colombo (EC -12-061) and the University of Melbourne (#1750854.1) to collect data and for secondary analysis. Participants provided written informed consent.

### Exposure measure—Greenness

Greenness was assessed using the Normalized Difference Vegetation Index (NDVI) which is an estimate of the levels of green vegetation in an area. NDVI ranges in values from –1 to 1, with 1 indicating the highest intensity of greenness and values below 0 indicating water [25]. NDVI was calculated in Google Earth Engine using ‘Landsat 5 Tier 1’ surface reflectance data [26] and the following equation:

$$\frac{\left(NIR-R\right)}{\left(NIR+R\right)}$$

where NIR is the near infra-red band and R is the red band. ‘Landsat 5’ data have a resolution of 30m x 30m.

A custom script collated all imagery for the study area between 1 May 2011 and 30 April 2012 and calculated the annual median NDVI for each pixel [27]. These dates were chosen because they a) represent a year of data immediately prior to the first survey data collection in May 2012, and b) have data availability. Landsat 5 ended in early May 2012 and Landsat 8 did not start recording images until mid-April 2013 [28]. Since water may have wellbeing and health benefits, we visually checked all NDVI values less than zero, and removed these from the dataset prior to analysis if they represented water features.

Participants’ residential neighborhoods were estimated by calculating circular buffers around their residential address at multiple scales: 100 m, 400 m, 800 m, 1,600 m, and 2,000 m. Multiple scales were chosen because the relevant distance is unknown. The 100 m distance represents the area immediately around the home. The 2,000 m distance was chosen because a systematic review of the distance within which greenness best predicts physical health found that larger distances (i.e., 2,000 m) are most predictive of physical health [29]. The other distances are typically used in research investigating links between the outdoor environment and cardiovascular related outcomes [30–32].

The mean NDVI of all grid cells intersecting each of these residential buffers was calculated using the QGIS v.3.0 ‘Zonal Statistics’ tool. Thus, our mean NDVI measure captures all locations within the circular buffers including along roads, in private residences, and in farms. In other words, while our measure includes green spaces andparks, it is not a measure of green spaces or parks. All spatial analyses were conducted using geographic information system (GIS) software packages: ArcGIS v.10.6 [33] and QGIS v.3.0 [34].

### Outcome measure—Heart disease

Individuals were asked ‘Do you have a heart disease known to you?’. Responses to this question were recorded as ‘yes’ or ‘no’.

### Covariates

Covariates for the analysis included age, marital status, educational attainment, income, alcohol use, and smoking history. These were chosen based on known heart disease risk factors [35]. Age was collected as a continuous variable and then re-categorized into the following subgroups: 34 to 39, 40 to 44, 45 to 49, and 50 to 55 years. Marital status responses were categorized as married, single, or divorced/other. Educational attainment was categorized as less than grade 10, ‘ordinary’ (between grade 10 and 11), and advanced (grade 12 and above). Data on average monthly income was collected in Sri Lankan Rupees (SLR) (approximately 160 SLR = one US$) and categorized as <10,000, 10,000 to 25,000, >25,000 to 50,000, and >50,000. Information on health compromising behaviors including current smoking (current non-smoker vs current smoker) and current alcohol use (any versus no alcohol intake) were collected.

Length of roads within each buffer was also included as a covariate since presence of roads may confound the relationship between greenness and cardiovascular disease. Roads necessarily limit the amount of surrounding greenness and also increase the amount of air pollution, which is associated with greater risk of heart failure [36] cardiovascular disease [37], and cardiovascular mortality [38]. We calculated road length for each participant and each buffer using Open Street Map data downloaded in 2020.

### Statistical analyses

Descriptive analysis was conducted to describe sample characteristics. Chi-square tests were run to examine bi-variate associations between exposure, covariates and the outcome. Pearson correlations were calculated to assess the relationships between mean NDVI across different buffer sizes. Prior to modelling, NDVI was multiplied by 10 so that the regression model coefficients can be interpreted as the effect of a 0.1 increase in mean NDVI. Multivariable logistic regression models were fitted to examine the association between mean NDVI scores within each buffer (100 m, 400 m, 800 m, 1,600 m, 2,000 m) and self-reported heart disease. The first model (Model 1) reported unadjusted odds ratios for the association. Model 2 fully adjusted for all covariates and confounders. All statistical analyses were carried out in statistical software STATA v.15.0 [39].

## Results

Of the population surveyed, 8.0% reported a diagnosis of heart disease (Table 1). The highest percentage of those with self-reported heart disease was found among those in the age group of 45 to 49 years (11.8%) and those who were single (9.3%) and with an advanced level of education (12.1%). Self-reported heart diseases were highest among those in the income group between 25,000 to 50,000. While the heart disease rate was unexpectedly lower for smokers and those who consumed alcohol, these differences were not statistically significant (smoking: *X*^2^ = 0.740, p = 0.390; alcohol: *X*^2^ = 2.233, p = 0.135).

**Table 1:** Sample characteristics (n = 5,268).

	Number	%	No heart disease %	Heart disease %
Age categories (years)				
34–39*	1,670	31.7	95.6	4.4
40–44	1,104	21.0	93.6	6.4
45–49	1,113	21.1	88.2	11.8
50–55	1,381	26.2	89.4	10.6
Marital status				
Married*	5,133	97.4	92.0	8.0
Not married	135	2.6	91.9	8.2
Education				
Advanced (grade 12+)*	1,195	22.7	88.0	12.1
Ordinary’ (grade 10–11)	2,036	38.7	94.7	5.4
<Grade 10	2,037	38.7	91.8	8.3
Income (Sri Lankan Rupees)				
>50 000	79	1.5	89.9	10.1
25 000–50 000	1,078	20.5	87.5	12.5
10 000–25 000*	3,193	60.6	93.7	6.3
<10 000	918	17.4	91.5	8.5
Alcohol				
No*	2,519	47.8	91.4	8.6
Yes	2,749	52.2	92.5	7.5
Smoking				
No*	3,771	71.6	91.8	8.2
Yes	1,497	28.4	92.5	7.5
Heart disease				
No	4,847	92.0		
Yes	421	8.0		

*Category used as reference group in regression models.

Mean NDVI was 0.7 across all buffers (Table 2). This means that on average, participants resided in areas with high vegetation cover. As a reference, high NDVI values are 0.6 to 0.9 and correspond to dense vegetation (e.g., forests or crops at the peak of growth) [40]. While NDVI was consistently high across the study area, there was variation in greenness with mean NDVI ranging from 0.00 to 0.84 for the 100 m buffer, and from 0.55 to 0.78 for the 2,000 m buffer.

**Table 2:** Characteristics of NDVI (1,600 m buffer) and self-reported heart disease.

	No heart disease		Heart disease	
	Mean	Std Dev	Mean	Std Dev
Mean NDVI – 100 m buffer	0.71	0.07	0.70	0.07
Mean NDVI – 400 m buffer	0.71	0.05	0.70	0.05
Mean NDVI – 800 m buffer	0.71	0.05	0.70	0.05
Mean NDVI – 1,600 m buffer	0.71	0.04	0.70	0.04
Mean NDVI – 2,000 m buffer	0.71	0.04	0.71	0.04

The percentage of participants with heart disease was highest in the least green quintile (11.4%) and lowest in the highest/greenest NDVI quintile (6.6%). A similar pattern was found for the 400 m and 800 m buffers with a lower percentage of participants with heart disease residing in the greener areas (results not reported).

Table 3 shows that the mean NDVI across the different buffers are positively correlated. As expected, mean NDVI is more strongly correlated when buffers are a similar size. For instance, the mean NDVI calculated within 1,600 m is almost perfectly correlated with the NDVI calculated within 2,000 m (*r* = 0.99). In contrast, the mean NDVI within 100 m is only moderately correlated with mean NDVI within 2,000 m (*r* = 0.69).

**Table 3:** Pearson correlations between mean NDVI across different buffers.

	100 m	400 m	800 m	1,600 m	2,000 m
**100 m**	1.00				
**400 m**	0.63	1.00			
**800 m**	0.51	0.88	1.00		
**1,600 m**	0.38	0.73	0.90	1.00	
**2,000 m**	0.36	0.69	0.86	0.99	1.00

Table 4 presents the odds ratios (OR) and 95% confidence intervals (CI) for Models 1 and 2 for each buffer size. The effect sizes indicate that a 0.1 increase in mean NDVI was associated with decreased odds of heart disease at the 400 m, 800 m, and 2,000 m scales. However, the confidence intervals were wide.

**Table 4:** Associations between mean NDVI buffer and self-reported heart disease across multiple buffers.

	Model 1		Model 2	
Mean NDVI	OR	95% CI	OR	95% CI
100 m	1.01	0.87, 1.16	1.01	0.87, 1.16
400 m	0.85	0.68, 1.05	0.80	0.64, 1.00
800 m	0.85	0.65, 1.12	0.85	0.63, 1.14
1,600 m	0.86	0.60, 1.25	0.93	0.64, 1.36
2,000 m	0.82	0.55, 1.24	0.74	0.48, 1.13

OR = odds ratio, CI = confidence interval.

Model 1 UnadjustedModel 2 Age + Marital Status + Income + Education + Alcohol + Smoking + Road Length.

## Discussion

Our study investigated associations between residential greenness and heart disease in a LMIC. We found a protective association between greenness within the 400 m, 800 m, and 2,000 m residential buffers and self-reported heart disease in working-age Sri Lankan men in fully adjusted models. This finding was notable, since our study area–an agricultural town in a tropical country–was predominantly green, as demonstrated by the moderate to high NDVI scores.

### Interpretation considering previous studies

While associations between residential greenness and cardiovascular related outcomes have not always been detected (e.g., [41]), our findings align with building evidence of the protective effect of residential greenness on cardiovascular related outcomes across temperate high and middle income countries [5,7,42–44]. Our study is one of a handful of recent studies that have investigated whether greenness has similar protective benefits in broader contexts; knowledge which is essential to inform land use planning and health promotion efforts in rapidly urbanising LMIC countries with high NCD burdens [45]. Similar to our findings, a multi-country study in Sub-Saharan Africa also found that residential greenness was beneficial to cardiometabolic health. Specifically, in rural, peri-urban, and urban centres a 0.1 unit increase in NDVI was associated with lower BMI, and lower odds of overweight/obesity, diabetes and allostatic load [13]. It is important to note here that both studies detected effects with a 0.1 unit increase in NDVI, which represents a feasible increase in greenness. Another example from Rio de Janeiro, Brazil–a tropical, highly urbanized, middle-income city–found that mortality rates for ischemic heart and cerebrovascular diseases were inversely associated with greenness [46].

Most studies of greenness and cardiovascular-related outcomes have explored variations in greenness around residential addresses or areas. However, there is now also evidence of beneficial effects at country level, with greener countries (including LMICs) having consistently lower prevalence of stroke and ischemic heart disease [47].Potential mechanisms explaining our findings

When considering evidence from different contexts, it is important to consider how the mechanisms and explanations for observed relationships may differ. As such, it is important to interpret our results considering what ‘green’ means in our study area where higher mean NDVI indicates denser forest, and greater distance from main roads, shops, and agricultural areas. Notably, there were no public parks or sports fields in our study area, and while agricultural areas are green, they are less green than forest. This is a substantially different context from most existing studies of greenness and health, and we need to take this into account when considering the potential mechanisms that might explain our findings.

Markevych and colleagues proposed three main pathways by which greenness could influence health: restoration (e.g., reduced stress, improved mental health), harm mitigation (e.g., reducing air pollution, noise, and heat), and instoration (e.g., supporting improved physical activity and social connection) [48]. While it is possible that greener areas in Ingiriya could be related to reduced risk of heart disease via reduced stress, we do not yet know whether the small differences in green that we observed result in discernible physiological differences. Some experimental studies have found that being exposed to green areas (viewing photos or visiting different areas) has short-term beneficial effects on objectively measured stress and cardiovascular biomarkers such as cortisol, heart rate and blood pressure [49–52]. However, to date, these studies have tended to be in higher income, temperate contexts, and have only compared relatively large differences in greenness, for example comparing photos of urban areas versus forests, or comparing visits to urban areas versus urban parks. Yet the differences in greenness in our study are far smaller than in these studies. Therefore, while it is possible that these physiological impacts may also occur with smaller differences in greenness, further experimental and observational research is needed to confirm this.

Harm mitigation via reduction of air pollution and noise may also explain why participants in greener areas had lower odds of heart disease [53]. Both air pollution and noise are linked to heart disease [37,54]. While we adjusted for road length, a proxy for both air and noise pollution, this does not completely capture all air and noise pollution [55,56]. Further, there is an implicit assumption that more roads equate to more traffic. It is likely, therefore, that we have not completely accounted for air pollution and noise. As such, our observed relationship between greenness and heart disease may be partially explained by unaccounted for harm mitigation.

Green spaces such as parks and sporting fields, are linked to higher levels of physical activity [57], with physical inactivity being a key risk factor for heart disease. Green spaces have also been linked to greater levels of social capital, cohesion and connectedness [58], with emerging evidence linking social cohesion to biomarkers of heart health [59]. However, this evidence is predominantly from urban areas in high income temperate countries, and, perhaps more importantly, parks and sports fields did not exist in our study area. In other words, the types of green spaces that were present in Ingiriya (dense forest, agricultural, gardens/street green) are not obviously linked to social factors and this is unlikely to explain our findings.

Study strengths and limitationsStrengths of our study include the use of a comprehensive census of men aged between 34 to 55 years residing in the study area, precise residential locations measured with GPS, and greenness measured objectively with satellite data. The study also benefited from a high response rate.

There were four key limitations relating to methodological and contextual issues. First, our exposure and outcome measures lacked specificity. We relied on self-reported heart disease status. This may have underestimated the prevalence of heart disease as some men may have had an undiagnosed heart disease, and this may be more likely in rural areas. Further, it is likely that men with higher socio-economic status were more likely to have had the opportunity to have heart disease diagnosed. Our measure of greenness did not distinguish between different types of vegetation and their relative benefits. For instance, our greenness measure did not differentiate between crops and other vegetation (e.g., trees in gardens or public areas), which may have different impacts on heart disease. Second, there was little variation in residential greenness in the study sample and all participants lived in areas that were at least moderately vegetated. In a study of green space and cardiovascular disease mortality in New Zealand, no significant effects were detected [60]. The authors suggested that this could have been because New Zealand, like Sri Lanka, also has abundant vegetation and greenness. Third, we did not have information on air quality of the areas. Since air pollution is linked with cardiovascular disease [37], and vegetation has been shown to remove air pollution [61], air pollution could be an unmeasured confounding factor in the association between greenness and heart disease. Finally, since this was a secondary analysis, our sample was restricted to working-age men and therefore may not be generalisable to other population groups.

Considering the discussion points above, our findings should be considered preliminary. Further research is needed to confirm and expand on these results. Ideally this would include objective measures of cardiovascular health, and more comprehensive assessments of environmental characteristics and detailed behaviors (e.g., physical activity levels and location, use of spaces with more vegetation).

## Conclusion

Our study provided new evidence of the relationship between greenness and heart disease in a rural area of tropical lower-middle-income country. We found higher residential greenness was associated with lower self-reported heart disease in working-age men. While our study area is not necessarily representative of Sri Lanka, or LMICs, and further research is needed to confirm our findings in similar contexts, our findings have added to emerging evidence highlighting the potential health benefits of residential greenness in LMICs.
